# Presumed Perinatal Ischemic Left Middle Cerebral Artery Stroke With Cerebral Palsy, Developmental Delay, and Epilepsy: A Case Report

**DOI:** 10.7759/cureus.66337

**Published:** 2024-08-06

**Authors:** Harneet S Randhawa, Jasneet Randhawa, Karanvir s Aulakh, Akshay More, Akshay Jain

**Affiliations:** 1 Radiology, Sassoon General Hospital, Pune, IND; 2 Radiology, Government Medical College, Baramati, IND; 3 Cardiology, Park Slope Cardiology, Brooklyn, USA; 4 Medicine, Aulakh Hospital, Amritsar, IND; 5 Medicine, Aulakh Bones and Joints Center, Tarn Taran, IND; 6 Interventional Radiology, Lokmanya Tilak Municipal Medical College and General Hospital, Mumbai, IND; 7 Radiology, Government Medical College, Kolhapur, IND

**Keywords:** epilepsy, cerebral palsy, developmental delay, presumed perinatal stroke, perinatal stroke

## Abstract

The perinatal period is a high risk for ischemic events to occur leading to lifelong morbidity. Various patterns of ischemic injury to the fetal and neonatal brain have been studied depending on gestational age as well as the degree of hypoxia/ischemia. We present a case of presumed perinatal ischemic left middle cerebral artery stroke diagnosed by magnetic resonance imaging (MRI) in a child with global developmental delay, cerebral palsy, and epilepsy. Interestingly, the typical features of middle cerebral artery stroke are often not present in perinatal strokes, and hence these are not imaged perinatally. Since studies and research into neuroplasticity and neuromodulation are current topics of interest and several research studies are being conducted, we wish to add this case to the available scientific literature.

## Introduction

The perinatal period is a high-risk period for the development of cerebrovascular accidents with about six times higher risk than in older children, and most of these are ischemic rather than hemorrhagic [1]. A cerebrovascular accident that occurs after 20 weeks of gestation and before 28 days of life is referred to as a perinatal stroke. Most cases of in-utero and neonatal stroke may not present with symptoms warranting thorough neuroimaging. Most neonates do not appear severely ill or may have mild symptoms or focal seizures [2].

Perinatal stroke can be divided into an acute symptomatic stroke or presumed perinatal stroke. Acute symptomatic stroke can further be classified into an acute ischemic stroke or acute hemorrhagic stroke. Presumed perinatal stroke does not present with acute symptoms of stroke and is thus detected later in life on neuroimaging for symptoms, such as developmental delay, seizures, and cerebral palsy. Presumed perinatal stroke is further classified into presumed perinatal ischemic stroke (PPIS) and presumed perinatal hemorrhagic stroke [3].

Perinatal arterial ischemic events have an estimated incidence of one in 2500 to one in 5000. Various maternal and fetal risk factors are responsible for such high incidence in the perinatal period. Maternal factors include primiparity, prothrombotic conditions, maternal substance use, pre-eclampsia, maternal diabetes mellitus, maternal thyroid disorders, prolonged labor, and intrauterine infections (chorioamnionitis by group B streptococcus or *E. coli*). Fetal factors include abnormalities of the cord, abnormalities of the placenta, fetal heart anomalies, respiratory and other causes of distress at birth, and immature vascular system [4].

Most perinatal strokes involve anterior circulation and the left hemisphere, with the left middle cerebral artery territory being the most common. The exact cause of the left-side predominance of PPIS is not clear. Direct origin of the left common carotid from the aorta along with the fetal circulatory system, patent ductus arteriosus, and foramen ovale have been postulated as possible culprits allowing embolus if any to reach the left side [5].

Contralateral hemiplegic cerebral palsy is a common motor outcome of perinatal middle cerebral artery strokes, associated with variable degrees of neurodevelopmental delay, sensory loss, and epilepsy. However, many patients have been shown to retain/regain several motor functions despite the loss of hemispherical areas responsible for controlling them; this has been attributed to developmental neuroplasticity [3].

Neuroplasticity after perinatal stroke is being increasingly studied. It has been described that at birth upper motor neurons innervate both sides of the body, i.e., have both ipsilateral and contralateral projections. During normal development, ipsilateral motor projections are lost, and contralateral motor projections mature. However, in patients with perinatal stroke, these ipsilateral pathways may persist and are responsible for the retention of some motor functioning. Moreover, these changes in neurodevelopment can be targeted by therapeutic rehabilitation. Therapeutic rehabilitation can be manual therapies or neuromodulation [3].

Manual therapies include constraint-induced manual therapy (CIMT) and bimanual training (BIT). In CIMT, the limb with normal functioning is restrained and the affected side use is promoted [6]. Bimanual therapy uses both limbs for skilled tasks [7]. Even though large-scale studies are needed to compare CIMT and BIT, a randomized control trial conducted by Hasan et al. in 2022 showed a statistically significant benefit of CIMT over BIT [8]. Neuromuscular electrical stimulation (NMES) includes transcutaneous stimulation of nerves or muscles to produce contraction [3,9]. Therapeutic neuromodulation includes transcranial magnetic stimulation (TMS) and transcranial direct current stimulation (TDS) using low-voltage electrical currents. These have also shown promising results and are under trial for cerebral palsy due to perinatal stroke [3,7]. However, large-scale randomized studies are needed to further strengthen the evidence of benefits. Neuroimaging especially tractography and functional magnetic resonance imaging (fMRI) have shown areas of interest that can be better targeted by these newer approaches [3].

## Case presentation

We present a case of a nine-year-old female child with global developmental delay, hemiplegic cerebral palsy, and epilepsy who presented to our radiology department for a brain MRI.

Birth history

The patient was born at 35 weeks of gestation by cesarean section due to intrauterine growth restriction and non-re-assuring fetal heart rate (FHR). Pregnancy was complicated by pre-eclampsia. At birth, the patient’s vitals and APGAR scores were normal and did not require neonatal ICU admission. The neuro-sonogram before discharge did not show any germinal matrix hemorrhage or features of periventricular leukomalacia.

Early development

Both gross and fine motor functions were delayed. The patient started standing at around 15 months and walking with support at around 24 months. The gait has become more stable over time. The patient had developed a hand preference for the left hand around one year of life.

The first seizure episode was noted at three years of age, which was a generalized tonic-clonic seizure (GTCS), for which the patient has been taking antiepileptics and had a stable course until recently when the patient developed an episode of status epilepticus while trying to wean off of antiepileptics. After aborting the episode and stabilizing the patient, she was referred to our tertiary care center by their peripheral practitioner.

Examination findings suggested gait abnormalities, reduced sensation on the right half of the body, and motor power was also slightly decreased on the right (3/5 in the right upper limb and 4/5 in the right lower limb). The left side had normal motor strength (5/5) and sensations.

MRI findings

**Figure 1:**
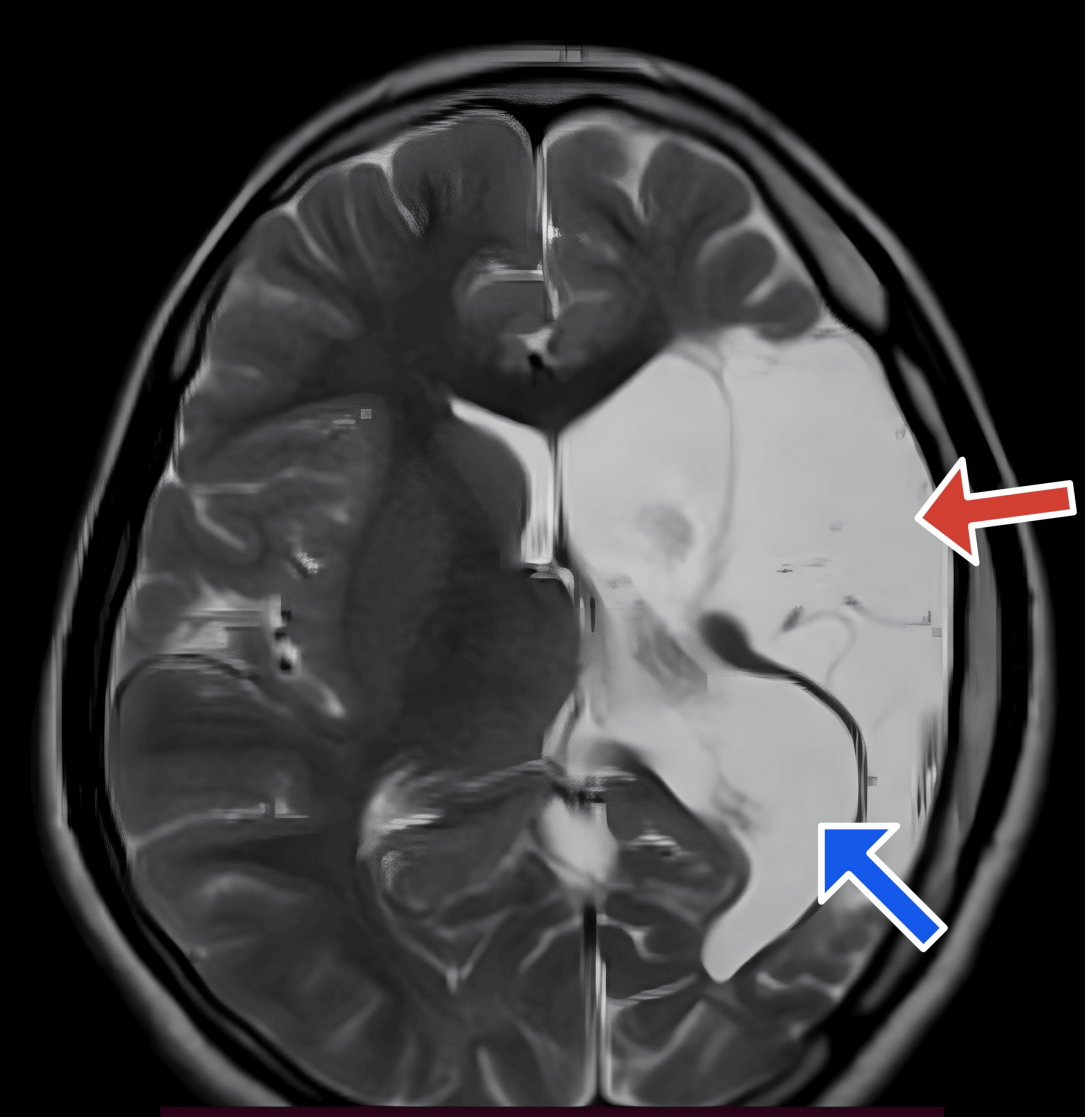
Axial T2 weighted image Cystic encephalomalacia (red arrow) in the left frontoparietal region with loss of white matter and ex-vacuo dilatation of ipsilateral lateral ventricle (blue arrow).

**Figure 2:**
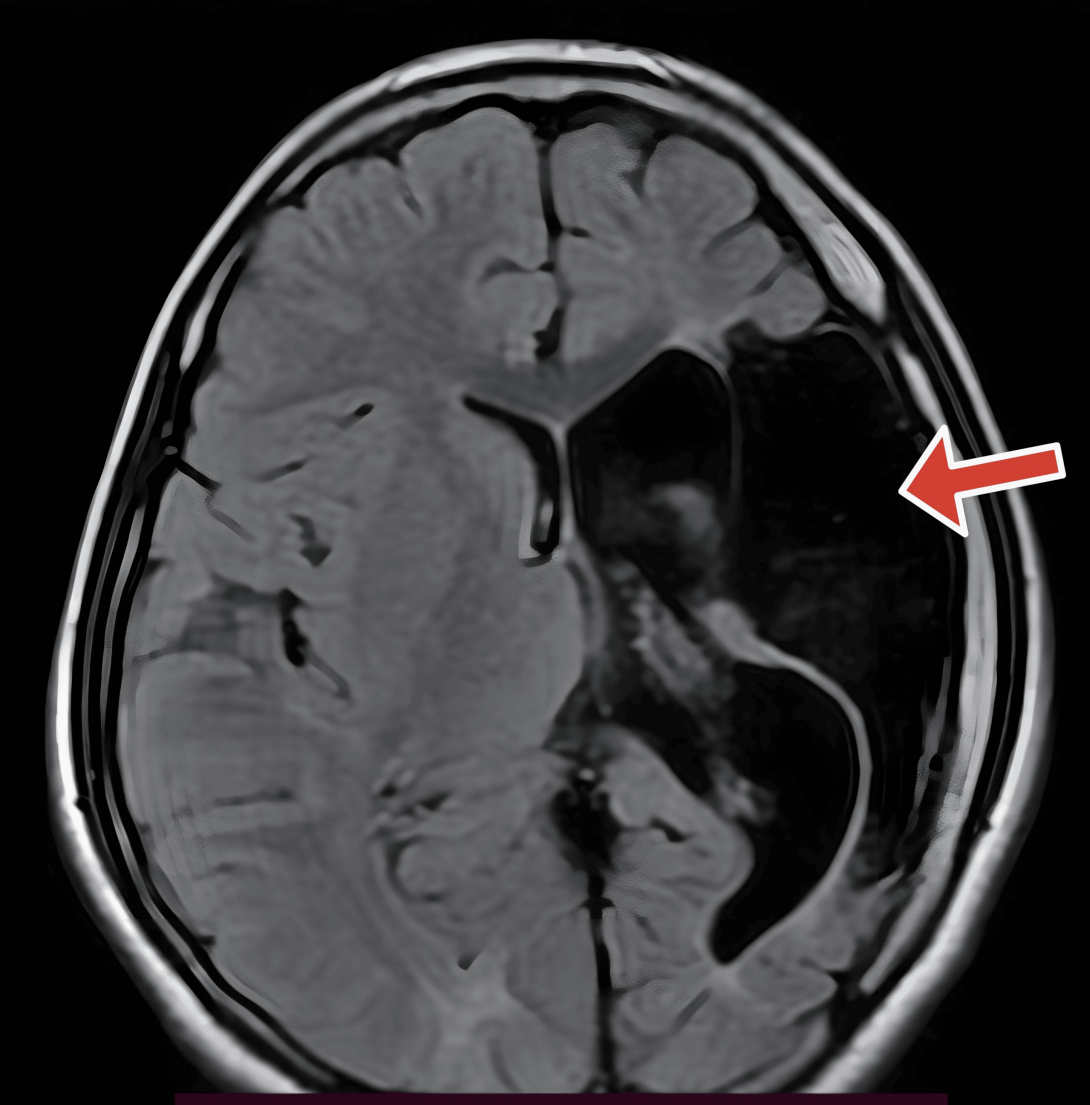
Axial T2 flair image Cystic encephalomalacia with suppression of fluid (red arrow).

**Figure 3:**
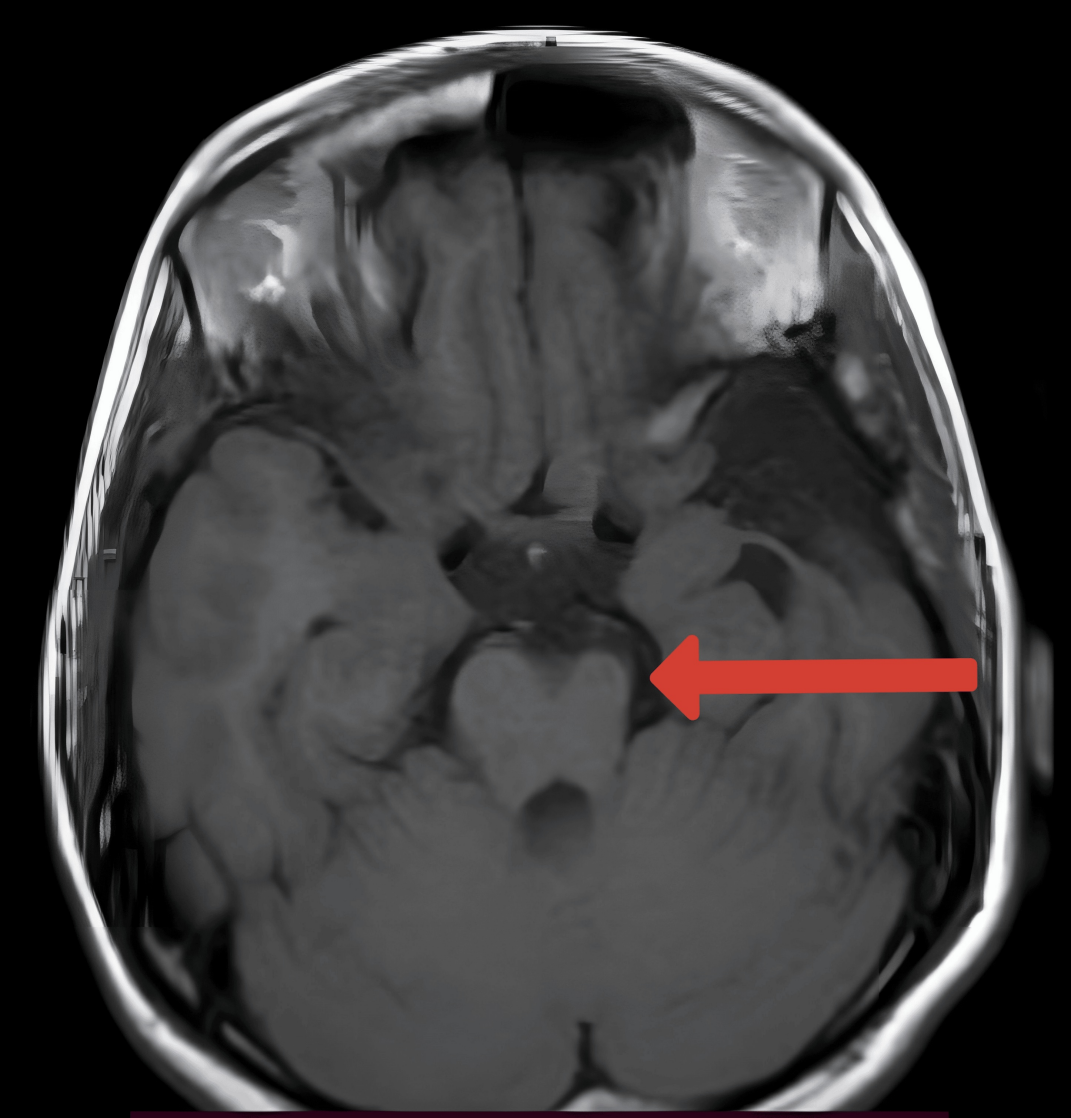
Axial T1 weighted image Atrophied left cerebral peduncle (red arrow).

**Figure 4:**
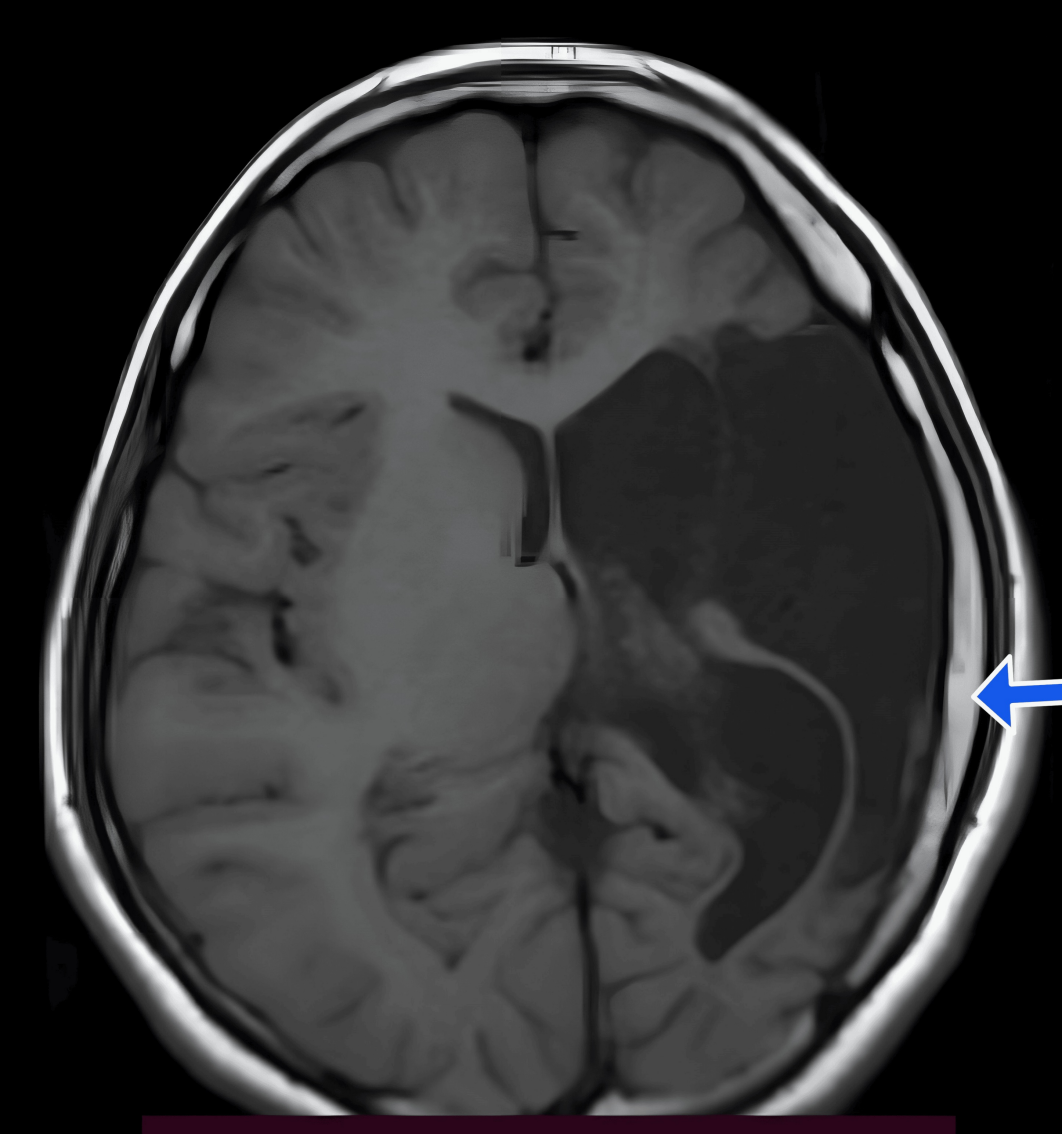
Axial T1 weighted image Ipsilateral calvarial thickening (blue arrow).

Multi-cystic encephalomalacia with adjacent gliosis was noted in the left frontal, parietal, and temporal regions (Figures 1, 2). Significant white matter loss and ex-vacuo dilatation of the ipsilateral lateral ventricle were also present (Figures 1, 2). An atrophied left cerebral peduncle suggestive of Wallerian degeneration was noted (Figure 3). Ipsilateral calvarial thickening was noted (Figure 4). No acute changes, diffusion restriction, bleeding, or calcification were present.

Considering the history of non-reassuring FHR requiring lower uterine segment section (LSCS) and no evidence of acute symptomatic presentation after birth, the diagnosis of PPIS involving the left MCA was made. The patient and her parents were counseled for proper antiepileptic treatment and rehabilitation by the physician before discharge.

## Discussion

PPIS is an ischemic stroke morphology detected on neuroimaging later in life for epilepsy, cerebral palsy, developmental delay, and others. It does not present acutely in the neonatal period thus most patients are not imaged in early [3]. In our case, the patient had no acute features of a stroke at birth and was discharged after a routine workup. Now the patient had come with seizures, cerebral palsy, and developmental delay with MRI suggesting a past stroke, i.e., PPIS involving the left middle cerebral artery.

Most patients with PPIS have mild symptoms and often develop epilepsy and developmental delays. Typical hemiplegia/paresis is less common; however, perinatal stroke is the most common cause of hemiplegic cerebral palsy. In our case, the patient only had mild motor weakness on the affected side and could walk without support despite the extensive loss of motor area as seen on the MRI. This can be explained by the model of neuroplasticity and ipsilateral upper motor neuron persistence in patients with perinatal stroke [3].

The most common vascular territory involved in PPIS is left MCA; the exact etiology for such involvement is not sure but some theories suggest that a fetal circulation with patent ductus arteriosus and foramen ovale may have a role [5]. Our patient had multi-cystic encephalomalacia involving the left frontal, parietal, and temporal regions with adjacent gliosis and ex-vacuo dilatation of the ipsilateral lateral ventricle. In keeping with the history of non-reassuring FHR requiring preterm caesarian, these imaging findings can be attributed to PPIS.

Our patient received treatment for seizures but not for motor weakness. Various manual and neuromodulation rehabilitation options are now available, which are helpful in such patients to improve motor functions [3]. The advantage of CIMT over BIT has been described by Hasan et al. [8]. However, large-scale randomized trials and factorial trials are needed to study the relative effectiveness of all interventions. Manual and neuromodulation rehabilitation especially if implemented early may help regain significant motor functioning and quality of life [3,7]. Early detection and treatment may have reduced the motor weakness and improved the gait early in our patient. Imaging should be offered to all patients as the earliest signs of cerebral palsy or confirmed developmental delay appear. Parental education about the same and government schemes to provide such facilities at discounted prices can be helpful.

Various studies have shown the significant utility of MRI in developmental delay and recommend it for underlying etiology detection, especially with tractography and fMRI, which can guide neuromodulation rehabilitation therapies [3,10]. Unfortunately, our patient was from a remote region and lacked early access to such facilities.

## Conclusions

Perinatal stroke may present atypically with mild to no clinical features suggestive of an acute cerebrovascular accident. Therefore, a high index of suspicion should be kept in all patients exposed to perinatal hypoxia or non-reassuring FHR. Infants with in-utero risk factors for perinatal ischemic insult should be closely monitored for signs of cerebral palsy or developmental delay and neuroimaging should be advised for the detection of PPIS/perinatal stroke if signs and symptoms are noted. Manual rehabilitation and neuromodulation therapies have shown improved quality of life in patients with perinatal stroke and cerebral palsy, especially with early intervention. Longitudinal studies involving early neuroimaging in high-risk patients are needed to guide clinical guidelines for imaging in patients with high suspicion of perinatal stroke. 
